# Association between antidementia medication use and mortality in people diagnosed with dementia with Lewy bodies in the UK: A retrospective cohort study

**DOI:** 10.1371/journal.pmed.1004124

**Published:** 2022-12-06

**Authors:** Shanquan Chen, Annabel C. Price, Rudolf N. Cardinal, Sinéad Moylett, Anne D. Kershenbaum, James Fitzgerald, Christoph Mueller, Robert Stewart, John T. O’Brien

**Affiliations:** 1 Department of Psychiatry, University of Cambridge, Cambridge, United Kingdom; 2 Cambridgeshire and Peterborough NHS Foundation Trust, Cambridge, United Kingdom; 3 Laboratory of Neuroimmunology, KU Leuven, Leuven, Belgium; 4 King’s College London, London, United Kingdom; 5 South London and Maudsley NHS Foundation Trust, London, United Kingdom; University of California San Francisco, UNITED STATES

## Abstract

**Background:**

Dementia with Lewy bodies (DLBs) is a common cause of dementia but has higher mortality than Alzheimer’s disease (AD). The reasons for this are unclear, but antidementia drugs (including acetylcholinesterase inhibitors [AChEIs] and memantine) symptomatically benefit people with DLB and might improve outcomes. We investigated whether AChEIs and/or memantine were associated with reduced hospital admissions and mortality.

**Methods and findings:**

We performed a retrospective cohort study of those diagnosed with DLB between 1 January 2005 and 31 December 2019, using data from electronic clinical records of secondary care mental health services in Cambridgeshire and Peterborough NHS Foundation Trust (CPFT), United Kingdom (catchment area population approximately 0.86 million), as well as linked records from national Hospital Episode Statistics (HES) data. Eligible patients were those who started AChEIs or memantine within 3 months of their diagnosis (cases) and those who never used AChEIs or memantine (controls). Outcomes included admission, length of stay, and mortality. Cox proportional hazard and linear regression models were used.

Of 592 patients with DLB, 219 never took AChEIs or memantine, 100 took AChEIs only, and 273 took both AChEIs and memantine. The cohorts were followed up for an average of 896 days, 981 days, and 1,004 days, respectively. There were no significant differences in the cohorts’ baseline characteristics, except for socioeconomic status that was lower in patients who never took AChEIs or memantine (*χ*^2^ = 23.34, *P* = 0.003). After controlling for confounding by sociodemographic factors (age, sex, marital status, ethnicity, socioeconomic status), antipsychotic use, antidepressant use, cognitive status, physical comorbidity, anticholinergic burden, and global health performance, compared with patients who never took AChEIs or memantine, patients taking AChEIs only or taking both had a significantly lower risk of death (adjusted hazard ratio (HR) = 0.67, 95% CI = 0.48 to 0.93, *p* = 0.02; adjusted HR = 0.64, 95% CI = 0.50 to 0.83, *P* = 0.001, respectively). Those taking AChEIs or both AChEIs and memantine had significantly shorter periods of unplanned hospital admission for physical disorders (adjusted coefficient −13.48, 95% CI = [−26.87, −0.09], *P* = 0.049; adjusted coefficient −14.21, 95% CI = [−24.58, −3.85], *P* = 0.007, respectively), but no difference in length of stay for planned admissions for physical disorders, or for admissions for mental health disorders. No significant additional associations of memantine on admission, length of stay, and mortality were found (all *P* > 0.05). The main limitation was that this was a naturalistic study and possible confounds cannot be fully controlled, and there may be selection bias resulting from nonrandom prescription behaviour in clinical practice. However, we mimicked the intention-to-treat design of clinical trials, and the majority of baseline characters were balanced between cohorts. In addition, our series of sensitivity analyses confirmed the consistency of our results.

**Conclusion:**

In this study, we observed that use of AChEIs with or without memantine in DLB was associated with shorter duration of hospital admissions and decreased risk of mortality. Although our study was naturalistic, it supports further the use of AChEIs in DLB.

## Author summary

**Why was this study done?:**

- Compared to Alzheimer’s disease (AD), dementia with Lewy bodies (DLBs) is associated with accelerated cognitive decline, lower quality of life, higher caregiver burden, shorter lifespan, and higher costs of care, as well as increased rates of admission to general hospitals and residential care and longer length of stay.
- Although there are no disease-modifying medications for DLB, there is evidence suggesting that acetylcholinesterase inhibitors (AChEIs) and glutamate N-methyl-D-aspartate receptor antagonists (specifically, memantine) are efficacious in DLB, though an impact on mortality is not clear.
- Distinct evidence on any survival benefit of AChEIs and memantine in DLB needs to be explored specifically, as DLB is pathologically and clinically different from AD and other forms of dementia.

**What did the researchers do and find?:**

- We identified 592 patients with DLB, including 219 who never took AChEIs or memantine, 100 who took AChEIs only, and 273 who took both AChEIs and memantine, between 1 January 2005 and 31 December 2019, using data from electronic clinical records of secondary care mental health services in Cambridgeshire and Peterborough NHS Foundation Trust (CPFT), United Kingdom and linked records from national Hospital Episode Statistics (HES) data.
- We investigated the associations of antidementia drug use in DLB with hospital admissions, length of stay, and mortality.
- We found that taking AChEIs alone or with memantine was associated with a significantly reduced risk of death and shorter hospital stays after unplanned admissions for physical disorders. Taking AChEIs alone or with memantine was not associated with the probability of admission for mental disorders or physical disorders (planned or unplanned), or the length of stay after admission for mental disorders or planned admission for physical disorders, compared to those not taking AChEIs or memantine. We found no significant additional association of memantine with admission, length of stay, or mortality, compared to those taking AChEI therapy alone.

**What do these findings mean?:**

- We observed that AChEIs (with or without memantine) may be associated with a shorter length of stay associated with unplanned hospital admissions for physical disorders and reduced risk of mortality, warranting further study.
- The findings in our study may help to address potential concerns that AChEIs could have the opposite effect (to increase mortality in DLB), thus giving both clinicians and people with DLB more evidence to support treatment decisions.
- The main limitation was that as a naturalistic study, possible confounds cannot be fully controlled and there may be selection bias resulting from nonrandom prescription behaviour in clinical practice. However, we mimicked the intention-to-treat design of clinical trials, and our series of sensitivity analyses confirmed the consistency of our results.

## Introduction

Dementia with Lewy bodies (DLBs) is the second most common form of neurodegenerative dementia after Alzheimer’s disease (AD) in people over 65 [1,2]. Together with Parkinson’s disease dementia (PDD), it constitutes Lewy body dementia (LBD). In a systematic review of 31 studies [1], the incidence of DLB was 3.8% of new dementia cases, and within the UK, DLB diagnostic rates were found to be 4.6% of all dementia cases [3].

Compared to AD, DLB is associated with accelerated cognitive decline, poorer quality of life, higher caregiver burden, higher costs of care, increased rates of hospital admission, longer hospital stays, and increased mortality (approximately twice that for AD) [2]. One study [4] estimated that in the United Kingdom, there are 80,000 people living with DLB who collectively experience >27,000 more hospitalizations per year than the same number of patients with AD, and spend >300,000 days more in hospital, costing >£35 million per year.

Interventions that could improve the prognosis of DLB even modestly would provide a major social and economic benefit for patients, their families, and the healthcare system. However, there are no disease-modifying therapies for DLB, and the management of DLB focuses on symptomatic control, largely based on the evidence base from AD and PDD. Data across all dementia [5], from AD [6–8] and from PDD [9], suggests an effect of acetylcholinesterase inhibitors (AChEIs) to reduce mortality; however, there is no such evidence for DLB despite its relatively high prevalence and poor prognosis. There is evidence in DLB, and in the broader group of LBD, of possible (though inconsistent) benefits of memantine for cognition and neuropsychiatric symptoms [10–13], but similarly no study has focused on any survival benefit. Evidence speaking to the impact of AChEIs and memantine on mortality is important, as DLB is pathologically and clinically different from, and has higher mortality than, AD and other forms of dementia.

People with DLB have complex physical and psychiatric symptoms and are more likely to be admitted to hospital than those without DLB. In this study, our primary aim was to investigate whether the use of AChEIs and/or memantine was associated with an altered risk of death in people with DLB. Our second aim was to investigate the association of AChEIs and/or memantine with the risk of hospital admission (planned or unplanned) and corresponding duration of hospital stay, for mental and physical disorders, in people with DLB. We hypothesised that both AChEIs and memantine would be associated with reduced risk of admission, decreased length of stay, and reduced mortality in people with DLB.

## Methods

### Study design and participants

We performed a retrospective cohort study using data from de-identified electronic clinical records of the secondary care mental health services of Cambridgeshire and Peterborough NHS Foundation Trust (CPFT), which provides specialist mental health and community physical health services to a population of approximately 0.86 million people in the United Kingdom. Therefore, all patients reported in CPFT were under the care of a specialist. CPFT’s electronic records contain patient information recorded during routine treatment, such as sociodemographic information, diagnosis, prescription data, death status, and clinical notes in free text. Clinicians enter aspects of this information in a systematic and structured/standard way to ensure its accuracy [14]. Data from patients with DLB were linked to national Hospital Episode Statistics (HES) data (from NHS Digital). Data were de-identified before researchers were given access [15,16].

**Fig 1:**
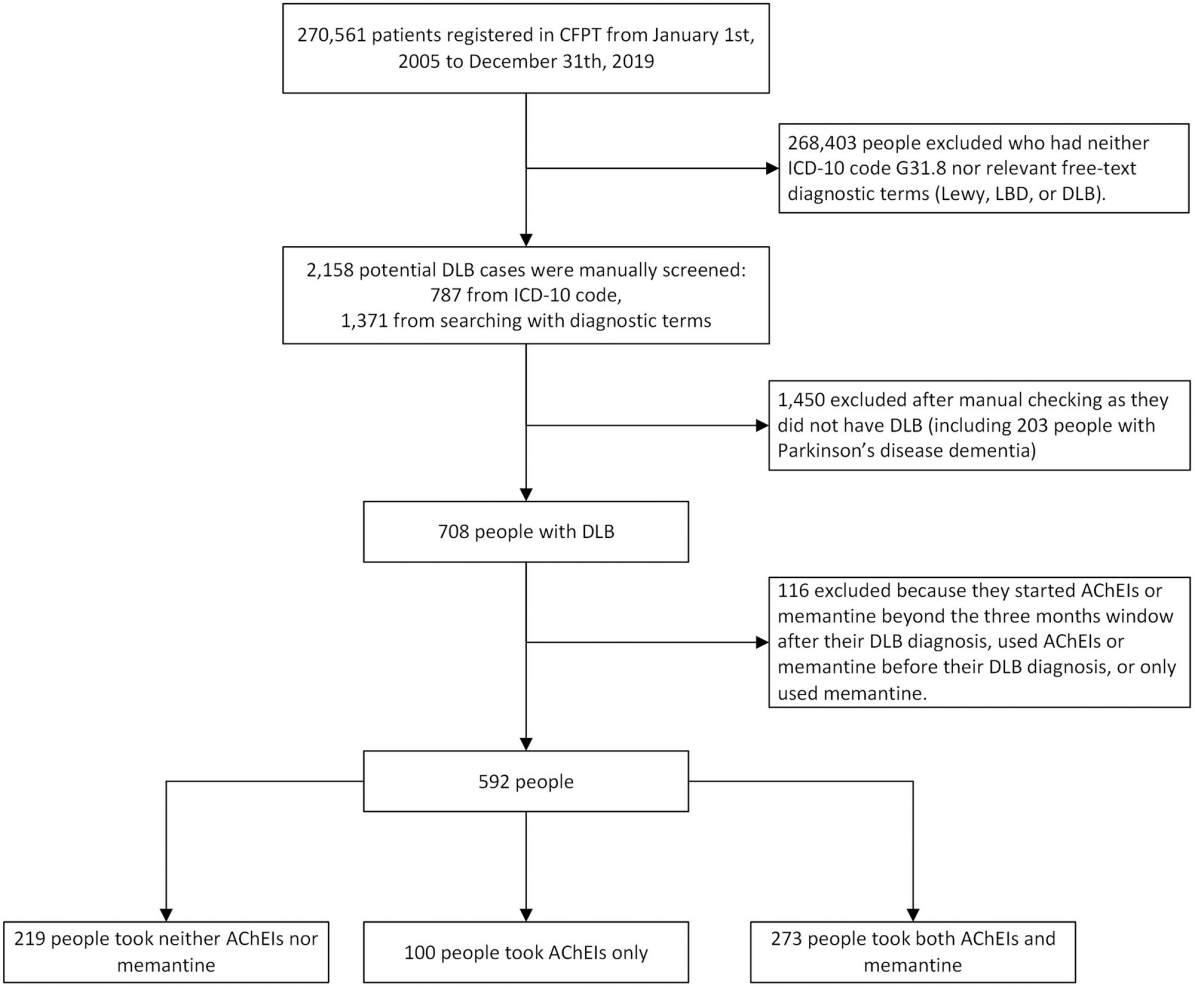
STROBE diagram showing construction of the cohorts. DLB, dementia with Lewy bodies; AChEIs, acetylcholinesterase inhibitors; LBD, Lewy body disease; DLB, dementia with Lewy body; CPFT, Cambridgeshire and Peterborough NHS Foundation Trust; ICD-10, World Health Organization International Statistical Classification of Diseases and Related Health Problems, 10th revision.

Data were collected from 1 January 2005 to 31 December 2019, to exclude the influence of the Coronavirus Disease 2019 (COVID-19) pandemic. Eligible patients were those diagnosed with DLB and who had at least 3 months’ follow-up (see below). DLB was established based on coded World Health Organization (WHO) International Classification of Diseases (ICD-10) diagnoses, as well as by searching de-identified free text for diagnostic terms. In detail, all patients with a clinician-recorded diagnostic code G31.8 or the presence of “Lewy,” “LBD,” and “DLB” in their assessment/diagnostic documents, progress notes, or clinical correspondence from the examining clinicians, were included as potential DLB cases. Then, all potential DLB cases were manually checked by 2 experienced dementia clinicians based on the DLB diagnosis guidelines [17]. The confirmed DLB cases thus selected were used for mortality studies in previous publications [14,18]. The procedure is summarised in Fig 1.

The index date was the date of diagnosis (for AChEIs and memantine non-users) or the first date of AChEIs or memantine prescription (for AChEIs or memantine users). Follow-up was until the patients’ final CPFT record date, the date of their death, or the study end date of 31 December 2019, whichever occurred first.

## Data collection

### Outcomes and measures

Admission information was acquired from the CPFT and HES databases, which also indicated whether admissions were planned or unplanned (for emergencies), were for mental disorders or for physical disorders, and gave the time of admission and discharge.

Length of stay was calculated as the sum of duration from admission to discharge for each admission during the follow-up. As the admission is a recurrent event, the longer a person was followed up, the higher probability of a longer total length of stay because of multi-admissions. To eliminate discrepancies due to differences in follow-up duration, the length of stay was standardised as the average annual length of stay per person per follow-up year, by calculating the total length of stay (days) during follow-up divided by the length of follow-up (days) and then multiplied by 365 (days).

Death status was ascertained by weekly linkage to national NHS Spine mortality data.

Medications. AChEIs were defined as donepezil hydrochloride, rivastigmine tartrate, and galantamine hydrobromide. Patients were defined as having been initiated on AChEIs or memantine if these were prescribed within 3 months after the DLB diagnosis. Patients who started AChEIs/memantine beyond this period after diagnosis, or used AChEIs/memantine before diagnosis, were excluded from analyses (Fig 1). This conservative definition was used in previous studies [6] and is intended to mimic the intention-to-treat design of clinical trials and to avoid reverse causation (in which possible adverse effects would cause changes in medication status). According to the UK National Institute for Health and Care Excellence (NICE) guideline, both AChEIs and memantine are licensed for the treatment of dementia; however, AChEIs are the first choice, so in clinical practice, it is uncommon to find patients who only receive memantine (21 cases). Also because our primary focus was on the AChEIs and few patients received only memantine, we excluded those who only received memantine (Fig 1). Thus, in this study, we categorised medication use as AChEIs only, a combination of AChEIs and memantine (AChEI+memantine), or neither/none.

### Covariates

We examined the following sociodemographic variables: age at baseline (years), sex (male versus female), marital status (married, cohabiting or civil partnership versus single, divorced or widowed), ethnicity (white versus other), and socioeconomic status (measured by the 2015 Index of Multiple Deprivation [IMD]). The IMD is the official measure of relative deprivation in England for a geographical area of residence and incorporates 7 domains: income, health and disability, barriers to housing and services, education, employment, crime, and living environment and education [19]. We also investigated physical co-morbidity (measured by the Charlson comorbidity index [CCI] [14], with co-morbid disorders identified via ICD codes in electronic medical records), use of antipsychotics, use of antidepressants, cognitive status (measured by a relative score of the Mini-Mental State Examination [MMSE], Montreal Cognitive Assessment [MoCA], or Addenbrooke’s Cognitive Examination III [ACE-III], closest to their index follow-up time), anticholinergic burden (measured by the anticholinergic cognitive burden [ACB] scale), and global health performance (measured by the Health of the Nation Outcomes Scales [HoNOS]). The CCI contains 19 categories of comorbidity and has been shown to be able predict 10-year mortality for patients who have a range of comorbid conditions [14]. The formula and ICD-10 codes used for identifying comorbidities can be found elsewhere [20,21]. Antipsychotics and antidepressants were selected according to UK NICE guidelines (Table A in S1 Appendix). Scores from 3 validated cognitive scales, the MMSE, MoCA, and ACE-III [22–24] were used, varying across different people according to clinical practice; we rescaled the scores to a consistent maximum of 100. The ACB scale was developed based on a systematic literature review of medicines with known anticholinergic activity [25]. In its newest (2012) version, the ACB scale included 115 medicines and scores each medicine 1, 2, or 3, based on their anticholinergic properties. The HoNOS is a clinician-rated instrument measuring the health and social functioning of patients [26]. It comprises 12 scales covering behaviour, impairment, symptoms, and social functioning, for which values are recorded ranging from 0 (no problem) to 4 (severe to very severe problem).

### Statistical analysis

For categorical variables, we have reported them as numbers (percentages) and continuous variables as mean (standard deviation, SD). Baseline differences between groups were assessed using ANOVAs (continuous variables) and chi-square tests (categorical variables).

Estimated projections of the cumulative hazard of admission and survival probability of mortality were obtained by Kaplan–Meier analysis. The log-rank test was used to determine whether survival curves differed statistically between individuals taking different medications.

The Cox proportional hazard model was used to estimate the hazard ratios (HRs) for medicine usage pattern in relation to admission or mortality. Results were reported as both unadjusted and adjusted hazard ratios (HRs). For adjusted HRs, the controlled covariates included sociodemographic factors (age, sex, marital status, ethnicity, socioeconomic status), antipsychotic use, antidepressants use, cognitive status, physical comorbidity, anticholinergic burden, and global health performance. As admission is a recurrent measure, the Cox model for admission was estimated by additionally using “person” as a cluster variable [27]. The proportional hazard assumption was tested as the correlation between the Schoenfeld residuals and survival time, with a significance level of *p* < 0.05 indicating nonproportionality. We also plotted the Schoenfeld residuals to test graphically the proportional hazard assumption (Fig A in S1 Appendix).

To analyse length of stay, we used linear regression, not Cox proportional hazard models. Linear regression was used to estimate both unadjusted and adjusted coefficients through which medicine pattern predicted length of stay. Controlled covariates were the same as that in the above Cox model.

To estimate the add-on association of memantine compared to those taking AChEIs only, we repeated the analyses above but taking “AChEIs only” as the reference.

Data were complete for outcomes and predictors except for cognitive scores. For this, we used multiple imputations with chained equations based on age, sex, marital status, ethnicity, CCI, IMD, antipsychotic use, and antidepressant use.

We conducted 4 sensitivity analyses. First, we repeated our primary analysis by propensity score weighting following a tutorial on propensity score estimation for multiple treatments [28]. In the propensity score estimation, our outcome was the medicine pattern and the predictors were the covariates controlled in the Cox model or linear regression. Second, we re-constructed the cohorts with a longer window, defining AChEI/memantine use as being within 6 months after the DLB diagnosis. Third, we repeated the analysis by setting the index date as the date of DLB diagnosis throughout in consideration of the start date being later for AChEI/memantine users, which may convey an automatic survival advantage. Fourth, we repeated the analysis by excluding the cognitive status in consideration of the missing and imputation of cognitive scores.

We used R (version 3.5.0) for all analyses and defined statistical significance as *P* < 0.05.

### Planning of analyses

No document analysis plan was created prior to the start of the study, although the analysis plan was discussed and agreed upon among co-authors. No data-driven changes to the analysis plan were made. One additional sensitivity analysis was included in response to peer review, by repeating our primary analysis using propensity score weighting.

This study is reported following the Strengthening the Reporting of Observational Studies in Epidemiology (STROBE) guideline (S1 STROBE Checklist) [29].

For identifiable records linkage, approval was obtained from the UK Confidentiality Advisory Group (CAG reference 18/CAG/0015) under section 251 of the UK National Health Service Act 2006. Data were de-identified before researchers were given access and analysed under NHS Research Ethics approvals (reference 18/EE/0029). Patients and carers have been involved throughout the process of cohort identification and data linkage that has allowed this analysis to take place. Patients and carers consent to the use of de-identified data for research purposes.

## Results

We identified 592 patients with the diagnosis of DLB, of whom 219 took neither AChEIs nor memantine, 100 took AChEIs only, and 273 took both AChEIs and memantine (Fig 1). The cohorts were followed up for an average of 896 days (range 30 to 3,736), 981 days (range 49 to 4,598), and 1,004 days (range 32 to 3,714), respectively (Table 1). There were no cohort differences in the baseline characteristics (Table 1), including age, sex, marital status, ethnicity, cognitive status, physical comorbidity, antipsychotic use, and antidepressant use (*p* > 0.05), except socioeconomic status (*p* = 0.003).

**Table 1:** Patient characteristics at baseline.

	None	AChEIs only	AChEIs + Memantine	Test statistic	*P*
	(*n* = 219)	(*n* = 100)	(*n* = 273)		
**Age (years) at baseline**	81.6 (7.87)	81.27 (7.14)	81.35 (7.35)	F = 0.07	0.93
**Sex (= male)**	112 (51.1%)	42 (42%)	148 (54.2%)	*χ*^2^ = 4.37	0.11
**Marital status (= married, cohabiting, or civil partnership)**	71 (32.4%)	42 (42%)	110 (40.3%)	*χ*^2^ = 4.17	0.12
**Ethnicity (= white)**	161 (73.5%)	77 (77%)	207 (75.8%)	*χ*^2^ = 0.56	0.75
**Socioeconomic status of area of residence (IMD) quintile**					
1 (most deprived)	21 (9.6%)	3 (3%)	33 (12.1%)		**0.003**
2	31 (14.2%)	23 (23%)	48 (17.6%)		
3	71 (32.6%)	21 (21%)	73 (26.7%)	*χ*^2^ = 23.34	
4	58 (26.6%)	21 (21%)	52 (19%)		
5 (least deprived)	37 (17%)	32 (32%)	67 (24.5%)		
**Cognitive status**	72.9 (18.1)	73.0 (20.8)	74.4 (19.5)	F = 0.42	0.65
**Physical comorbidity**	5.76 (2.32)	5.53 (2.25)	5.6 (2.44)	F = 0.41	0.67
**Antipsychotic use (= yes)**	51 (23.3%)	25 (25%)	70 (25.6%)	*χ*^2^ = 0.37	0.83
**Antidepressant use (= yes)**	83 (37.9%)	43 (43%)	106 (38.8%)	*χ*^2^ = 0.78	0.68
**Anticholinergic burden**	1.50 (1.54)	1.91 (2.53)	1.87 (2.16)	F = 2.42	0.09
**Global health performance**	11.5 (5.58)	11.63 (6.36)	11.86 (5.16)	F = 0.30	0.74
**Admission for mental disorders (= yes)**	7 (3.2%)	4 (4%)	21 (7.7%)	*χ*^2^ = 5.27	0.07
Frequency (times per person-year)	0.96 (1.03)	0.53 (0.3)	0.63 (0.46)	F = 1.22	0.31
Length of stay (days per year)	56.74 (65.88)	56.64 (49.06)	43.82 (42.56)	F = 0.33	0.72
**Admission for physical disorders, planned (= yes)**	56 (25.6%)	32 (32%)	95 (34.8)	*χ*^2^ = 4.91	0.09
Frequency (times per person-year)	1.05 (1.39)	0.74 (0.85)	0.71 (0.75)	F = 2.17	0.12
Length of stay (days per year)	16.16 (35.59)	16.26 (29.97)	13.7 (34.49)	F = 0.12	0.89
**Admission for physical disorders, unplanned (= yes)**	172 (78.5%)	79 (79%)	212 (77.7%)	*χ*^2^ = 0.10	0.95
Frequency (times per person-year)	1.61 (1.48)	1.47 (1.43)	1.31 (1.07)	F = 2.48	0.08
Length of stay (days per year)	36.09 (65.42)	22.92 (25.42)	22.18 (37.2)	F = 4.30	**0.01**
**Death (= yes)**	177 (80.8%)	65 (65%)	182 (66.7%)	*χ*^2^ **=** 14.58	**0.001**
**Follow-up duration (year)**	2.46 (2.14)	2.69 (1.97)	2.75 (1.8)	F = 1.43	0.24

Data are shown as mean (standard deviation) or number (%). *P* values for age, cognitive status, physical comorbidity, anticholinergic burden, global health performance, frequency of admission, length of stay, and follow-up duration were obtained by ANOVA, and for others via Pearson’s chi-square test. Socioeconomic status is measured by the IMD; cognitive status is measured by scales of MMSE, MoCA, or ACE-III, rescaled for each test to a maximum of 100. Physical comorbidity is measured by the CCI. Anticholinergic burden is measured by ACB scale. Global health performance is measured by Health of the Nation Outcomes Scales.

ACB, anticholinergic cognitive burden; ACE-III, Addenbrooke’s Cognitive Examination III; AChEIs, acetylcholinesterase inhibitors; CCI, Charlson comorbidity index; IMD, Index of Multiple Deprivation; MMSE, Mini-Mental State Examination; MoCA, Montreal Cognitive Assessment.

**Fig 2:**
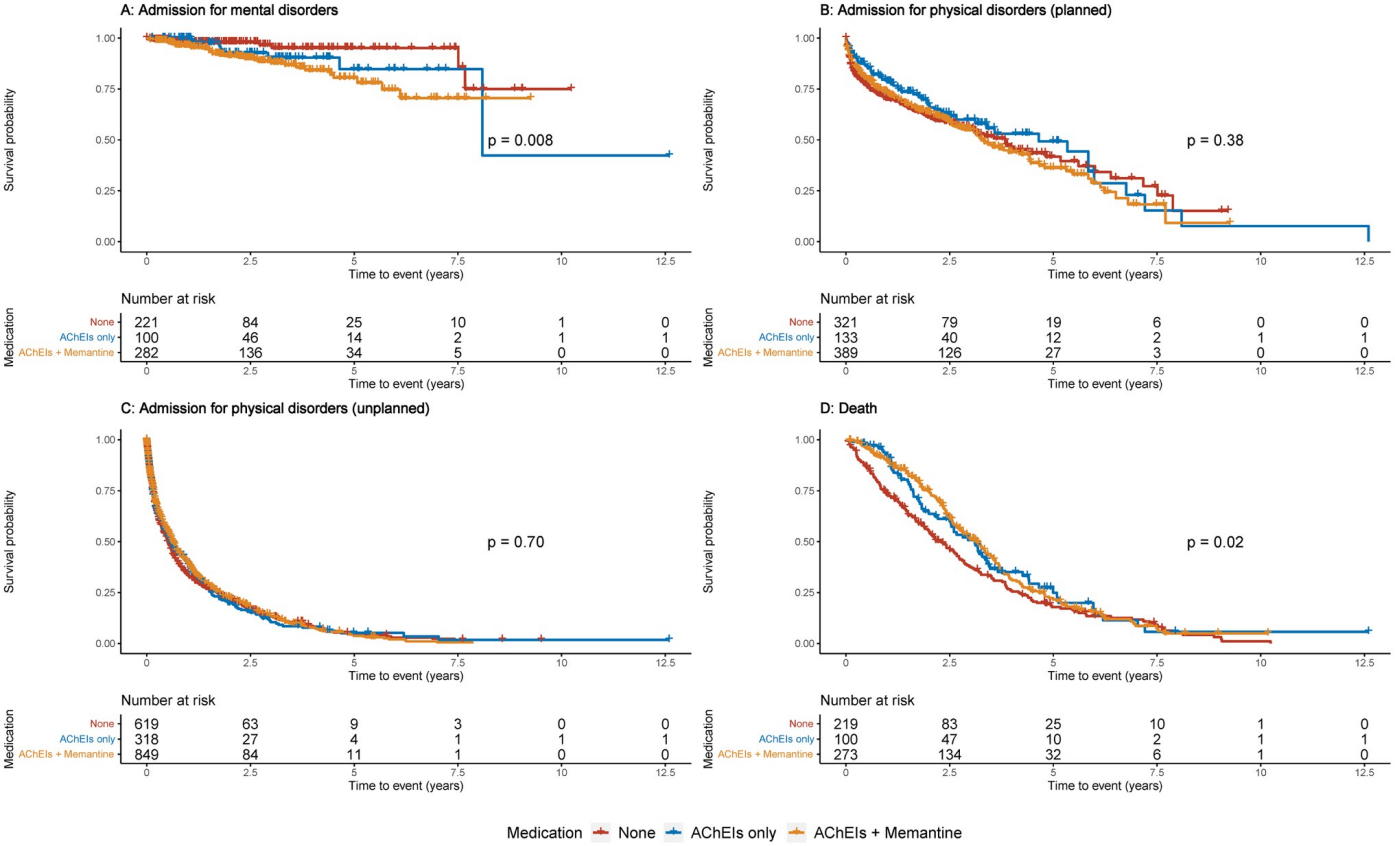
Kaplan-Meier survival curves of admissions and mortality in patients with dementia with Lewy bodies, by medication use. Panels A, B and C present the recurrent events. *P* values are calculated from the log-rank test. AChEIs, acetylcholinesterase inhibitors.

Fig 2 present the survival curves. In unadjusted analyses, mortality rates were lower in people taking AChEIs or AChEI+memantine than those taking neither (*p* = 0.02, Fig 2D), and the AChEI and AChEI+memantine groups had higher rates of admission for mental health disorders than those taking neither (*p* = 0.008, Fig 2A). There were no significant differences in the rates of planned admissions for physical disorders (*p* = 0.38, Fig 2B), or unplanned admissions for physical disorders (*p* = 0.70, Fig 2C).

**Fig 3:**
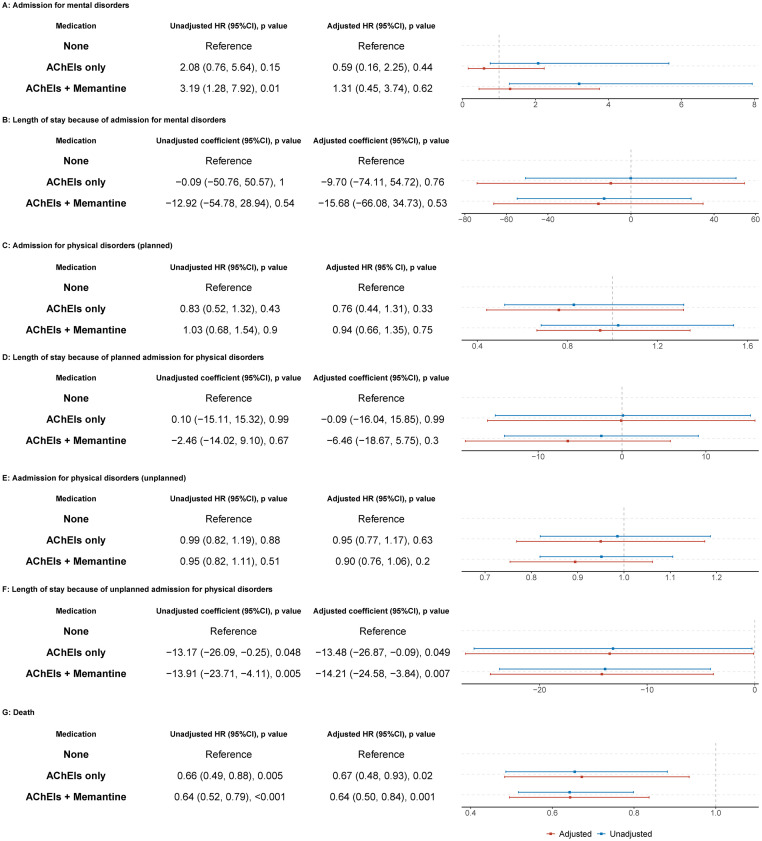
Association of antidementia medication use with risk of admission, length of stay, and risk of death among patients with dementia with Lewy bodies. Hazard ratios (HR), 95% confidence intervals (CI), and *p* values were estimated from Cox proportional hazards models. Coefficients, 95% confidence intervals (CI), and *p* values were estimated from linear regression. Adjusted HRs/coefficients were adjusted for age, sex, marital status, ethnicity, socio-economic status (Index of Multiple Deprivation), antipsychotic use, antidepressant use, cognitive status/score, physical comorbidity, anticholinergic burden, and global health performance. The blue lines show the unadjusted HRs/coefficients, the red lines show the adjusted ones, and the grey dotted line shows the cut-off for negative or positive associations (0 for coefficients and 1 for hazard ratios). AChEIs, acetylcholinesterase inhibitors.

However, after controlling for confounding by sociodemographic factors (age, sex, marital status, ethnicity, socioeconomic status), antipsychotic use, antidepressants use, cognitive status, physical comorbidity, anticholinergic burden, and global health performance, patients taking AChEIs only had a significantly lower risk of death than patients taking neither drug class (adjusted HR = 0.67, 95% CI = 0.48 to 0.93; *p* = 0.02, Fig 3G), and had shorter total hospital stays for unplanned admission for physical disorders (adjusted coefficient −13.48, 95% CI = [−26.87, −0.09]; *p* = 0.049, Fig 3F). Associations between AChEIs use and other outcomes were not significant, including the risk of admission for mental disorders (adjusted HR = 0.59 95% CI = 0.16 to 2.25; *p* = 0.44, Fig 3A), length of stay because of admission for mental disorders (adjusted coefficient −9.70, 95% CI = [−74.11, 54.72]; *p* = 0.76, Fig 3B), risk of planned admission for physical disorders (adjusted HR = 0.76 95% CI = 0.44 to 1.31; *p* = 0.33, Fig 3C), length of stay because of planned admission for physical disorders (adjusted coefficient −0.09, 95% CI = [−16.04, 15.85]; *p* = 0.99, Fig 3D), and risk of unplanned admission for physical disorders (adjusted HR = 0.95, 95% CI = 0.77 to 1.17; *p* = 0.63, Fig 3E). Similarly, patients taking both AChEIs and memantine had a significant lower risk of death (adjusted HR = 0.64, 95% CI = 0.50 to 0.84; *p* = 0.001, Fig 3G) and shorter stay because of unplanned admission for physical disorders (adjusted coefficient −14.21, 95% CI = [−24.58, −3.84]; *p* = 0.007, Fig 3F), but associations for other outcomes were not significant, including risk of admission for mental disorders (adjusted HR = 1.31, 95% CI = 0.45 to 3.74; *p* = 0.62, Fig 3A), length of stay because of admission for mental disorders (adjusted coefficient −15.68, 95% CI = [−66.08, 34.73]; *p* = 0.53, Fig 3B), risk of planned admission for physical disorders (adjusted HR = 0.94, 95% CI = 0.66 to 1.35; *p* = 0.75, Fig 3C), length of stay because of planned admission for physical disorders (adjusted coefficient −6.46, 95% CI = [−18.67, 5.75]; *p* = 0.30, Fig 3D), and risk of unplanned admission for physical disorders (adjusted HR = 0.90, 95% CI = 0.76 to 1.06; *p* = 0.20, Fig 3E). The proportional hazard assumption was valid for medicine usage pattern across outcomes (all *p* > 0.05, Fig A in S1 Appendix).

After controlling for confounding by sociodemographic factors (age, sex, marital status, ethnicity, socio-economic status), antipsychotic use, antidepressants use, cognitive status, physical comorbidity, anticholinergic burden, and global health performance, the differences between the AChEI+memantine group and the AChEI-only group were not significant for any of these outcomes (*p* > 0.05, Fig B in S1 Appendix).

Sensitivity analyses using propensity score weighting (Fig C in S1 Appendix), a longer initiation window for AChEIs/memantine (Fig D in S1 Appendix), setting the index date as the date of DLB diagnosis throughout (Fig E in S1 Appendix), and excluding cognitive status as a predictor (Fig F in S1 Appendix) all confirmed our primary results, that use of AChEIs with or without memantine in DLB was associated with decreased mortality and shorter total hospital stays for unplanned admission for physical disorders.

## Discussion

Using a retrospective cohort study based on a large and comprehensive electronic clinical records database, we investigated the associations of antidementia drug use in DLB with hospital admissions, length of stay, and mortality. Groups at baseline were similar in terms of age, sex, and cognitive test score. After controlling for sociodemographic factors, antipsychotic use, antidepressant use, cognitive status, and physical comorbidity, we found that patients who took AChEIs only, or took both AChEIs and memantine, had a significantly lower risk of death than those taking neither. Although no significant associations were found between use of AChEIs with or without memantine and the number of hospital admissions, the length of stay in patients taking AChEIs with or without memantine was significantly shorter for unplanned admissions for physical disorders. No significant additional associations of memantine over AChEIs were found.

Our findings supported the hypothesis that taking AChEIs is associated with a reduced risk of death in DLB. This association is consistent with the survival benefits of AChEIs identified in those with unspecified dementia [5], AD [6–8], and PDD [9]. Patients with AD, PDD, and DLB have severe deficits in brain levels of acetylcholine and its synthetic enzyme, choline acetyltransferase, and AChEIs are beneficial for improving related clinical symptoms by lowering degradation of acetylcholine once released into the synapse [30,31]. In addition, AChEI use in DLB can cause improvements in cognition, including attention, and improvements in neuropsychiatric features including visual hallucinations, a hallmark of the disorder. These benefits have led to AChEIs being recommended for use in DLB by national and international expert groups including the International DLB Consortium, the UK NICE, and the British Association for Psychopharmacology [17,32,33].

No significant associations were found between AChEIs usage and the risk of admissions (for mental disorders, or for planned or unplanned physical disorders). This inconsistency was unexpected given the significant association between AChEI use and lower risk of mortality identified in this study, the established evidence that AChEIs have been associated with improvements in global cognitive function, behavioural disturbances, activities of daily living, and overall function in people with LBD or DLB [11,13,34], and the considerable overlap between the primary death reason of DLB [35] and primary admission reason of DLB [4] or LBD [36]. Therefore, one of the possible mechanisms is that the symptomatic benefit of AChEIs could be translated into better physical health and then lower mortality by reducing events such as falls, but the absence of a significant association between AChEIs and admission (especially unplanned admission) might result from unmet needs or underutilised admission. For example, fall-related injuries and behavioural/neuropsychiatric symptoms of dementia accounted for about 13% of all admissions of DLB in South London, UK, far lower than the proportion (about 64%) observed for LBD in Florida, United States of America [4,36]. Another possible mechanism is that AChEIs have been directly associated with reductions in physical conditions. Growing studies support the beneficial role of AChEIs in the cardiovascular system and suggest that AChEIs have anti-inflammatory properties and reduce markers of endothelial and platelet activation [37–40]. Evidence from AD and all dementia spectrum also indicated that AChEIs is associated with reduced risk of stroke [5] and myocardial infarction [8]. Although an effect of AChEIs to improve cognitive function is well established, the possibility that AChEIs reduce mortality by improving the course of dementia itself should be treated cautiously, as the association between AChEI use and the onset of severe AD has not been found to be significant [6].

Taking AChEIs or AChEIs+memantine was associated with reduced unplanned admission days for physical disorders. This finding also supports AChEI usage in this group, given the substantial amount of time that was spent in hospital by people with DLB (Table 1).

We found no additional associations with respect to memantine. In relation to hospital admissions, this is to some extent consistent with another study reporting no significant association between memantine and nursing home admission in mild dementia (including DLB and AD) [41]. Meta-analysis and randomised controlled trials (RCTs) have found inconsistent but primarily negative findings that memantine improved [10] or did not improve [11–13] neuropsychiatric symptoms, improved quality of life [42], had no effect on general cognition [11], no effect on the clinical global impression of change [10], and no effect on motor symptoms [11] in DLB, versus placebo. There is also evidence from 1 meta-analysis [13] and an RCT study [43] indicating that AChEIs, but not memantine, significantly improved cognitive function, behavioural symptoms, and activities of daily living in DLB.

The findings in our study help to address potential concerns that the AChEIs could have the opposite effect and increase mortality in DLB by, for example, their propensity to cause bradycardia [44,45]. There are often concerns about the use of AChEI in DLB, including that long-term use may be associated with syncope, bradycardia, autonomic dysfunction, postural hypotension, and cardiac problems. It is therefore reassuring that we found mortality was lower in patients taking an AChEI, which do not support the association between AChEI use and negative long-term outcomes, thus giving both clinicians and people with DLB more evidence to support treatment decisions.

Strengths of our study included the use of an anonymised electronic records database derived from routinely collected clinical records enabling a larger sample size than many previously published examinations of DLB cohorts, also reducing sampling bias in relation to diagnosed cases [18]. Because we used routine NHS e-record data for a mental health trust covering a substantial geographical area and population, we can be confident our findings reflect antidementia drug use and outcomes for DLB within routine NHS services. We also adopted a comprehensive approach to identifying DLB cases, not just relying on ICD codes (which can be used inconsistently) but also identifying DLB cases by text searches of diagnostic terms followed by clinical validation. In addition, by using linked HES data, we were able to study the associations of drug use with admission and length of stay and to examine planned/unplanned admissions as well as admissions for mental/physical disorders.

Limitations include that as a naturalistic study, possible confounds cannot be fully controlled; as this is not a RCT, use of AChEIs and memantine is not random between cohorts. In practice, medicines may be more likely to be prescribed to healthy patients, and the use of medicines with anticholinergic effects may also affect the prescription of AChEIs. In addition, unequal presence of adverse events of AChEIs (like syncope, bradycardia, autonomic dysfunction, postural hypotension, and cardiac problems), which may also function as contra-indications for starting AChEIs, may also have resulted in relevant selection bias. However, we sought to mimic the intention-to-treat design of clinical trials, excluding those who used AChEIs before the index date, and the majority of baseline characters (including global health performance and anticholinergic burden) were balanced between cohorts. Despite this, we conducted a sensitivity analysis with propensity score weighting, and the results remained consistent.

A second limitation is the conservative design in which we defined medication exposure as being within the 3 months after DLB diagnosis. Patients who were defined as taking AChEIs only, or AChEIs+memantine, might have stopped taking 1 or both medications (e.g., due to adverse effects), but would still be analysed within their starting cohort. On one hand, this limitation could attenuate the association in question if it takes prolonged exposure for AChEIs and/or memantine to work. On the other hand, this limitation could also lead to overestimation of this association if treatment of behavioural problems improved users’ awareness of the specific drug benefits, and such people receive more or are willing to take more antidementia medication. However, there is no evidence supporting such a possible cumulative effect. In addition, the sensitivity analysis with a longer drug initiation window confirmed our primary results.

Thirdly, although at baseline there were no significant differences in the use of antipsychotics and antidepressants among our cohorts, over time, there may have been a different pattern in groups of antidementia medication users. The possibility is that there may have less use of antipsychotics and antidepressants in those using antidementia medications, as these groups already have more polypharmacy. This may have an influence on the associations in which we were interested, when our models only controlled for antipsychotics and antidepressants at baseline as confounders. However, we reported both unadjusted and adjusted estimations, and both estimations were consistent.

Fourthly, we used scaled scores from multiple cognitive tests and imputed some missing cognitive data, which might have affected (underestimated or overestimated) associations of interest. However, results from Cox regressions with and without cognitive status were similar and robust.

Finally, because this was a sample based on clinical diagnosis, we were unable to determine how much the association with mortality was driven by those with mixed AD/DLB pathology, compared to more “pure” AD cases, since concurrent AD pathology has been shown to be associated with poorer outcome in some studies on DLB [46–48].

One unanswered question from this work is the dose–response relationship between AChEIs and the outcomes we examined. Previous evidence indicates that higher doses of AChEIs appear to lead to better survival benefits in patients with AD [6] and better improvement on neuropsychiatric symptoms in patients with DLB [11]. Additionally, we did not examine specific AChEIs. There are differences between donepezil, rivastigmine, and galantamine in the magnitude of the association with mortality in patients with AD [6] and in associations with neuropsychiatric symptoms and general cognition in patients with DLB [11]. One study suggested a possible cutoff dose for these 3 AChEIs to reduce the risk of death in patients with AD [6]. However, we were unable to check these 2 questions, because our real-world data did not provide sufficient DLB cases with single-AChEI therapy, we lacked precise data on dosage, and patients’ medication dosages may have changed frequently. These unanswered questions require further study.

In patients with DLB, taking AChEIs (with or without memantine) was associated with a significantly reduced risk of death, and reduced length of hospital stay due to unplanned admissions for physical health problems. Our findings provided new evidence for the possible benefits of AChEI treatment for those with DLB and suggest corresponding RCT studies. In addition, further studies should investigate possible mechanisms for the benefit of increased survival.

## Supporting information

S1 Appendix Additional information on study methods and findings. (DOCX)

S1 STROBE Checklist Strengthening the reporting of observational studies in epidemiology (STROBE) checklist. (DOCX)

## Abbreviations

ACB: anticholinergic cognitive burden
ACE-III: Addenbrooke’s Cognitive Examination III
AChEIs: acetylcholinesterase inhibitors
AD: Alzheimer’s disease
CCI: Charlson comorbidity index
COVID-19: Coronavirus Disease 2019
CPFT: Cambridgeshire and Peterborough NHS Foundation Trust
DLB: dementia with Lewy body
HES: Hospital Episode Statistics
HoNOS: Health of the Nation Outcomes Scales
HR: hazard ratio
ICD: International Classification of Diseases
IMD: Index of Multiple Deprivation
LBD: Lewy body dementia
MMSE: Mini-Mental State Examination
MoCA: Montreal Cognitive Assessment
NICE: National Institute for Health and Care Excellence
PDD: Parkinson’s disease dementia
RCT: randomised controlled trial
SD: standard deviation
WHO: World Health Organization
