# Supplementary material for: Association between antidementia medication use and mortality in people diagnosed with dementia with Lewy bodies in the UK: A retrospective cohort study
Source: PLoS Med. 2022 Dec 6;19(12):e1004124. doi: 10.1371/journal.pmed.1004124 (PMC9725132; doi:10.1371/journal.pmed.1004124)

**Table A: List of medicines**

| Antipsychotics | **First-generation:** Benperidol, chlorpromazine, flupentixol, fluphenazine, haloperidol, levomepromazine, pericyazine, perphenazine, pimozide, pipotiazine, prochlorperazine, promazine, trifluoperazine, zuclopenthixol  **Second-generation:** Asenapine, amisulpride, aripiprazole, clozapine, iloperidone, lurasidone, olanzapine, paliperidone, quetiapine, risperidone, sertindole, sulpiride, ziprasidone, zotepine |
| --- | --- |
| Antidepressants | Agomelatine, amitriptyline, bupropion, citalopram, clomipramine, dosulepin, doxepin, duloxetine, escitalopram, fluoxetine, fluvoxamine, imipramine, isocarboxazid, lofepramine, maprotiline, mianserin, mirtazapine, moclobemide, nefazodone, nortriptyline, paroxetine, phenelzine, reboxetine, sertraline, tranylcypromine, trazodone, trimipramine, tryptophan, venlafaxine, vortioxetine |

**Fig A. Test of the proportional hazard assumption.** Data present the Schoenfeld residuals extracted from Cox proportional hazards models, with the corresponding variable (shown on the title in each panel) as the outcome and medicine pattern as the key predictor, controlling for age, sex, marital status, ethnicity, socio-economic status (Index of Multiple Deprivation), antipsychotic use, antidepressant use, cognitive status/score, physical comorbidity, anticholinergic burden, and global health performance. The p-value was extracted from the Schoenfeld test (via the function cox.zph in R) on the corresponding Cox model, with a significance level of p<0·05 indicating non-proportionality.


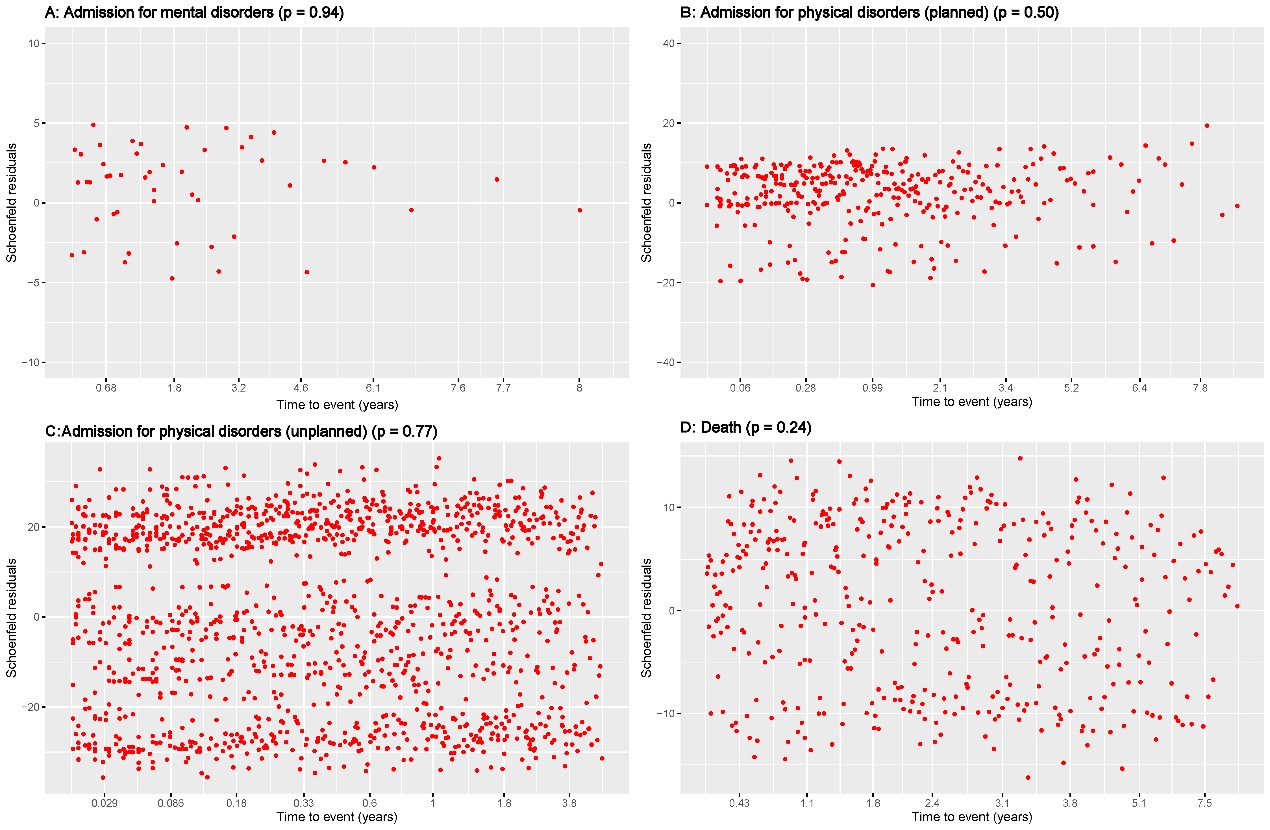


**Fig B. Comparison of patients with DLB taking AChEIs and memantine with those taking AChEIs alone, for risk of admission, length of stay, and risk of death.** Hazard ratios (HR), 95% confidence intervals (CI), and *p* values were estimated from Cox proportional hazards models. Coefficients, 95% confidence intervals (CI), and *p* values were estimated from linear regression. Adjusted HRs/coefficients were adjusted for age, sex, marital status, ethnicity, socio-economic status (Index of Multiple Deprivation), antipsychotic use, antidepressant use, cognitive status/score, physical comorbidity, anticholinergic burden, and global health performance. The blue lines show the unadjusted HRs/coefficients, the red lines show the adjusted ones, and the grey dotted line show the cut-off for negative or positive associations (0 for coefficients and 1 for hazard ratios). AChEIs, acetylcholinesterase inhibitors.


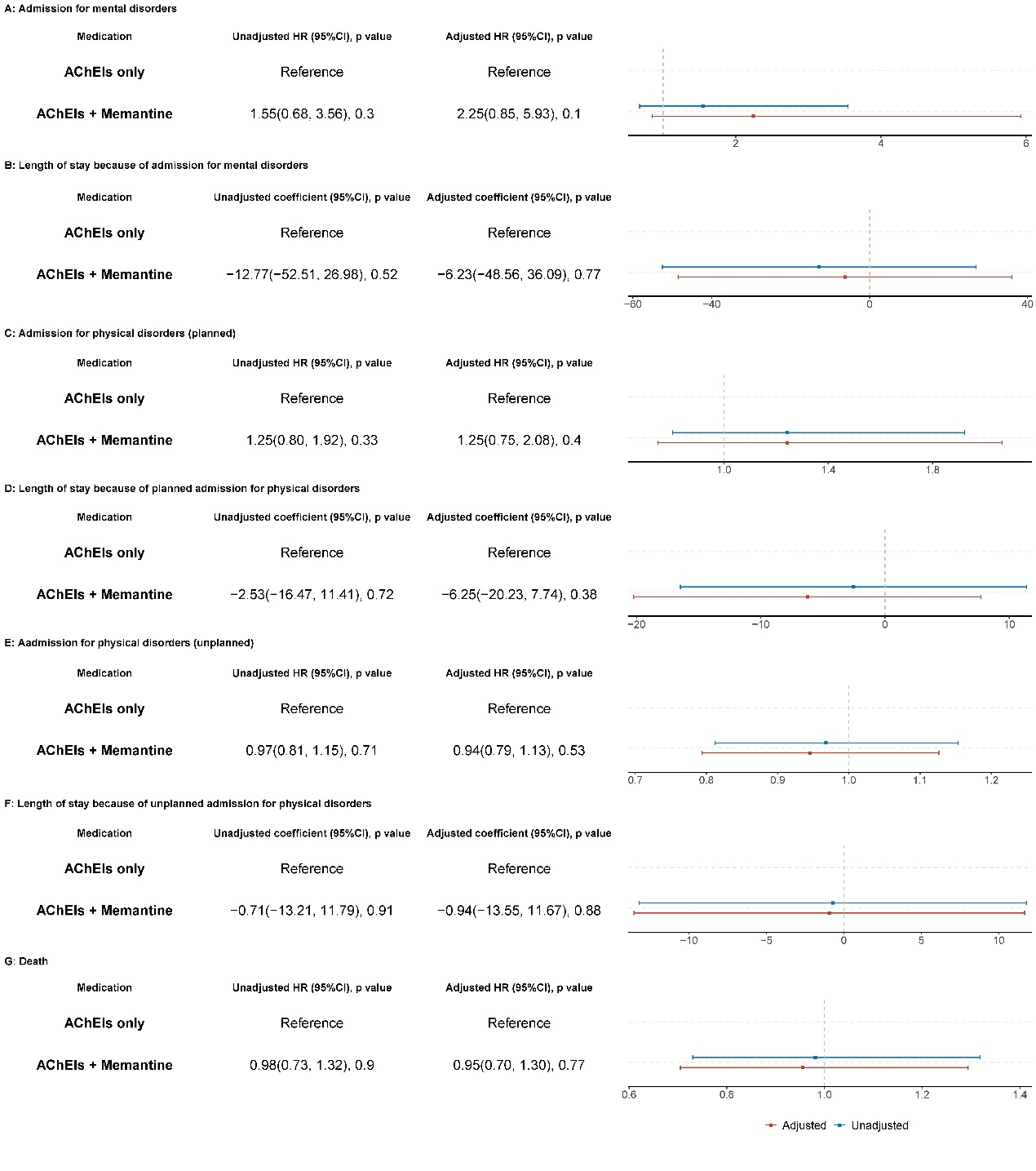


**Fig C. Association of antidementia medication use with risk of admission, length of stay, and risk of death among patients with DLB: sensitivity analysis by using propensity score weighting.** Hazard ratios (HR), 95% confidence intervals (CI), and *p* values were estimated from weighted Cox proportional hazards models. Coefficients, 95% confidence intervals (CI), and *p* values were estimated from weighted linear regression. Adjusted HRs/coefficients were adjusted for age, sex, marital status, ethnicity, socio-economic status (Index of Multiple Deprivation), antipsychotic use, antidepressant use, cognitive status/score, physical comorbidity, anticholinergic burden, and global health performance. In the propensity score estimation, our outcome was the medicine pattern and the predictors were the covariates controlled in the Cox model or linear regression; The blue lines show the unadjusted HRs/coefficients, the red lines show the adjusted ones, and the grey dotted line show the cut-off for negative or positive associations (0 for coefficients and 1 for hazard ratios). AChEIs, acetylcholinesterase inhibitors.


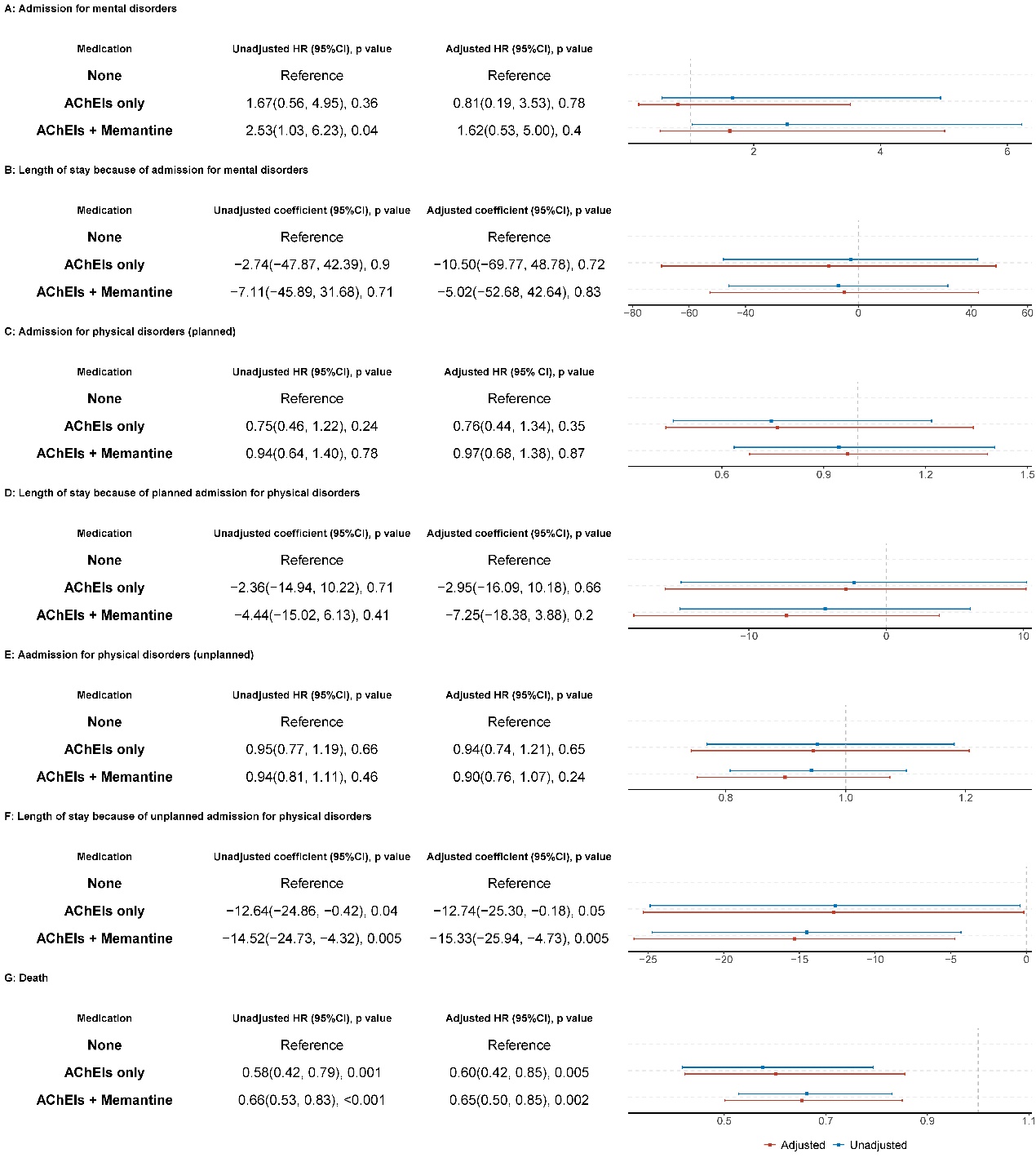


**Fig D. Association of antidementia medication use with risk of admission, length of stay, and risk of death among patients with DLB: sensitivity analysis with a 6 month window to define AChEI/memantine user****s.** Hazard ratios (HR), 95% confidence intervals (CI), and *p* values were estimated from Cox proportional hazards models. Coefficients, 95% confidence intervals (CI), and *p* values were estimated from linear regression. Adjusted HRs/coefficients were adjusted for age, sex, marital status, ethnicity, socio-economic status (Index of Multiple Deprivation), antipsychotic use, antidepressant use, cognitive status/score, physical comorbidity, anticholinergic burden, and global health performance. The blue lines show the unadjusted HRs/coefficients, the red lines show the adjusted ones, and the grey dotted line show the cut-off for negative or positive associations (0 for coefficients and 1 for hazard ratios). AChEIs, acetylcholinesterase inhibitors.


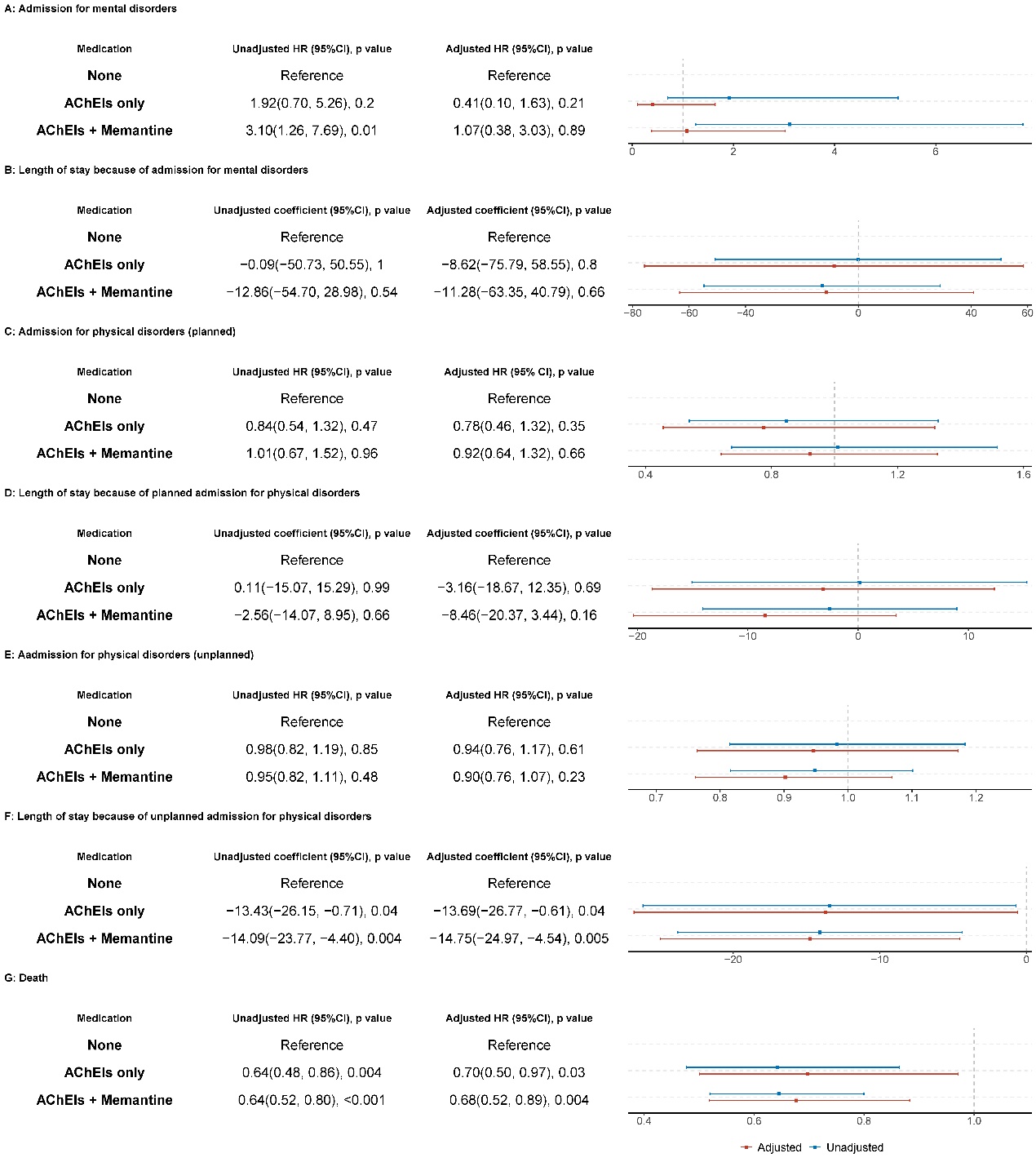


**Fig E. Association of antidementia medication use with risk of admission, length of stay, and risk of death among patients with DLB: sensitivity analysis by setting the index date as the date of DLB diagnosis throughout.** Hazard ratios (HR), 95% confidence intervals (CI), and *p* values were estimated from Cox proportional hazards models. Coefficients, 95% confidence intervals (CI), and *p* values were estimated from linear regression. Adjusted HRs/coefficients were adjusted for age, sex, marital status, ethnicity, socio-economic status (Index of Multiple Deprivation), antipsychotic use, antidepressant use, cognitive status/score, physical comorbidity, anticholinergic burden, and global health performance. The blue lines show the unadjusted HRs/coefficients, the red lines show the adjusted ones, and the grey dotted line show the cut-off for negative or positive associations (0 for coefficients and 1 for hazard ratios). AChEIs, acetylcholinesterase inhibitors.


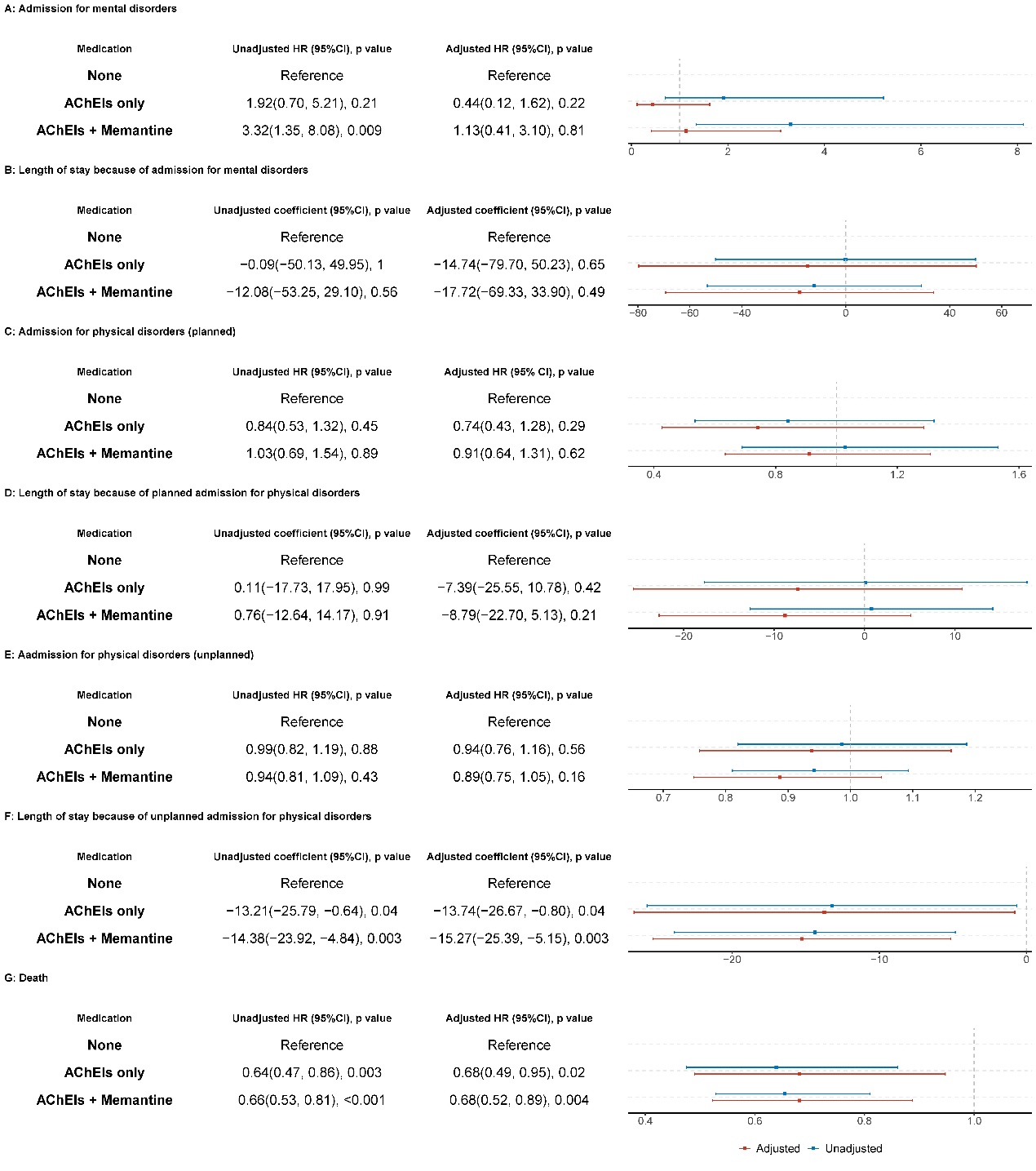


**Fig F. Association of antidementia medication use with risk of admission, length of stay, and risk of death among patients with DLB: sensitivity analysis by excluding the cognitive status variable.** Hazard ratios (HR), 95% confidence intervals (CI), and *p* values were estimated from Cox proportional hazards models. Coefficients, 95% confidence intervals (CI), and *p* values were estimated from linear regression. Adjusted HRs/coefficients were adjusted for age, sex, marital status, ethnicity, socio-economic status (Index of Multiple Deprivation), antipsychotic use, antidepressant use, cognitive status/score, physical comorbidity, anticholinergic burden, and global health performance. The blue lines show the unadjusted HRs/coefficients, the red lines show the adjusted ones, and the grey dotted line show the cut-off for negative or positive associations (0 for coefficients and 1 for hazard ratios). AChEIs, acetylcholinesterase inhibitors.


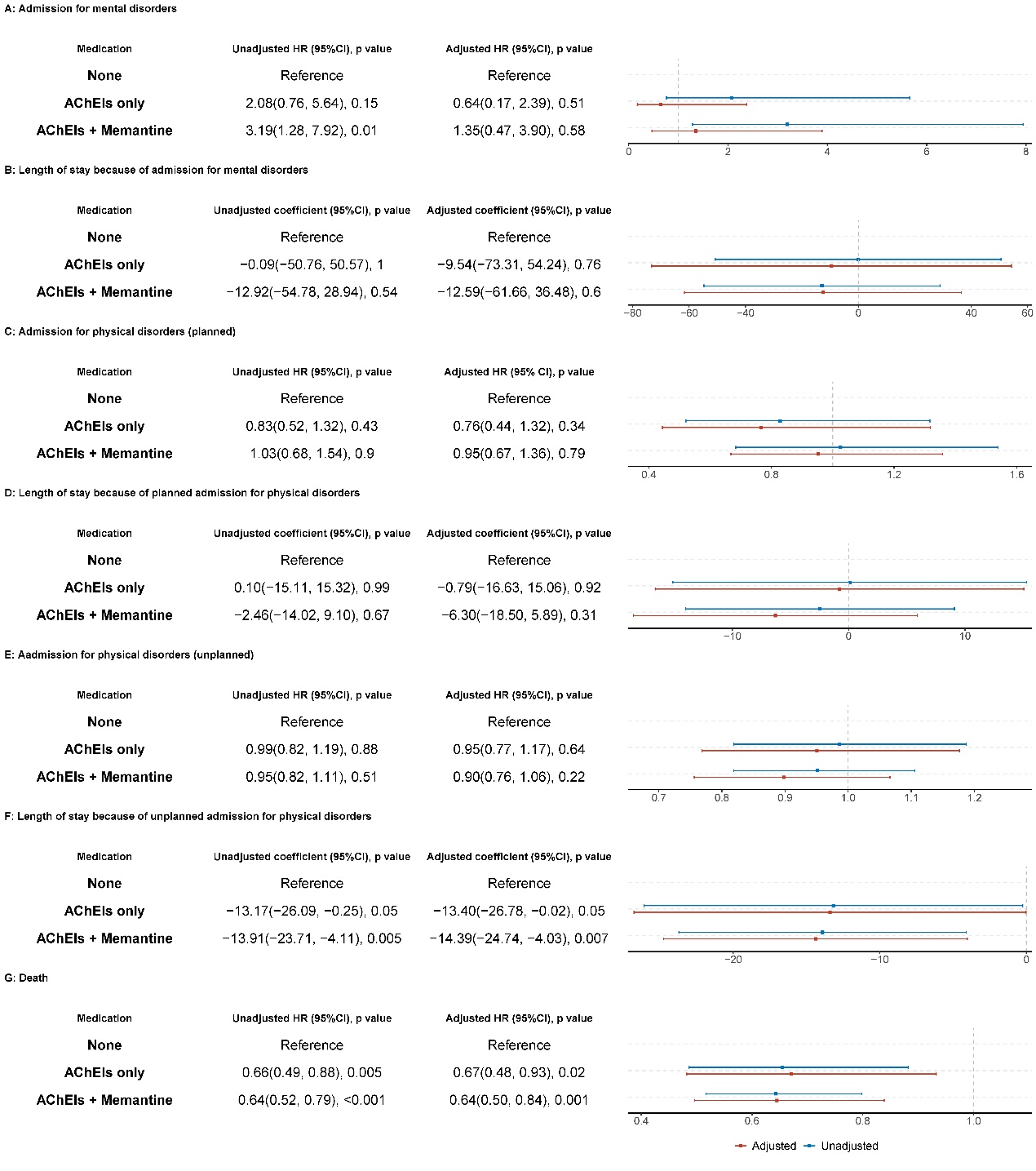

Supplement: S1 Appendix — (DOCX) [file pmed.1004124.s001.docx]
